# Predictive Value of MHR, PLR Combined With NLRP1 for Severity and Long‐Term Prognosis in Premature Coronary Artery Disease

**DOI:** 10.1002/iid3.70202

**Published:** 2025-05-19

**Authors:** Mengyun Zhu, Jianying Shen, Weijing Liu, Hui Sun, Yawei Xu

**Affiliations:** ^1^ Department of Cardiology Shanghai Tenth People's Hospital, Tongji University School of Medicine Shanghai China

**Keywords:** coronary artery severity, long‐term prognosis, MHR, NLRP1, PCHD, PLR

## Abstract

**Objective:**

To investigate the predictive value of platelet to lymphocyte ratio (PLR), monocyte to high‐density lipoprotein ratio (MHR) combined with nucleotide binding oligomeric domain like receptor protein 1 (NLRP1) for the severity of premature coronary heart disease (PCHD) and its 2‐year long‐term prognosis.

**Method:**

A total of 132 patients with PCHD examined in our hospital from February 2020 to January 2022 were retrospectively selected as the research objects. All patients who met the criteria were divided into mild group, moderate group, and severe group according to the severity of PCHD. The patients were followed up for 2 years. Patients were then divided into good prognosis group (without adverse cardiovascular events, *n* = 96) and poor prognosis group (with adverse cardiovascular events, *n* = 36). The predictive value was evaluated by ROC curve and multivariate logistic regression analysis.

**Results:**

Compared with the mild group, the levels of MHR, PLR, and NLRP1 in the moderate group and the severe group were significantly increased (*p* < 0.05). The levels of MHR, PLR, and NLRP1 in the poor prognosis group were higher than the good prognosis group (*p* < 0.05). The area under the curve (AUC) of MHR, PLR, and NLRP1 alone and in combination for predicting the 2‐year long‐term prognosis of patients was 0.787, 0.653, 0.869, and 0.926, respectively. Combined markers showed superior predictive accuracy (*p* < 0.05). After adjusting for confounding factors such as treatment, comorbidities, weight, gender, and smoking, MHR, PLR, and NLRP1 were independent risk factors for severe progression and poor prognosis of PCHD (*p* < 0.05).

**Conclusion:**

MHR, PLR, and NLRP1 were increased in patients with higher severity of PCHD and poor prognosis. The combined detection has certain clinical guiding value for PCHD. However, this study was a single‐center retrospective study with a small sample size. Thus, the results need to be further verified.

## Graphical abstract

CAD patients with high severity and poor prognosis had high content of MHR, PLR, and NLRP1. The combined detection of these indicators had certain clinical value for predicting the severity and long‐term prognosis of CAD.

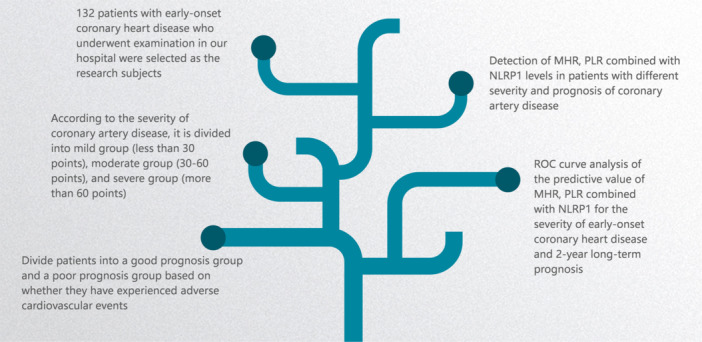

## 1 Introduction

Premature coronary heart disease (PCHD) is defined as a premature atherosclerotic disease with coronary artery stenosis of 70% or more or acute myocardial infarction (MI) before the age of 45 years. With the improvement of living conditions, the incidence of PCHD gradually increased, while the age of onset gradually decreased [1, 2]. Early diagnosis of PCHD can significantly reduce the occurrence of unstable PCHD, MI and other complications, and can significantly reduce the occurrence of cardiac‐related mortality. In the past, the diagnosis was mainly made by coronary angiography. However, coronary angiography is an invasive means. Although it is helpful for the diagnosis of PCHD, it may cause some complications for patients, and may not be suitable for all patients [3]. Therefore, it is necessary to find other simple, convenient, and effective indicators for the diagnosis of PCHD.

Inflammation is an important risk factor for the development of cardiovascular diseases. Studies have found that cardiovascular disease is more common in patients with high circulating inflammatory markers [4]. The platelet to lymphocyte ratio (PLR) is a simple and easy‐to‐calculate indicator of inflammation, which reflects the balance between platelets and lymphocytes. Platelets play a key role in the process of atherosclerosis and thrombosis, while lymphocytes are involved in immune regulation [5]. The monocyte to high‐density lipoprotein ratio (MHR) combines both monocyte and high‐density lipoprotein factors. Monocytes are the main source of anti‐inflammatory substances during atherogenesis, and HDL has anti‐atherogenic effects. The increase of MHR may reflect the imbalance between atherogenic and antiatherogenic, and thus become an important index to evaluate the risk of PCHD [6]. The study has shown that NLRP1 can activate Caspase‐1 and promote the secretion of inflammatory cytokines, thereby aggravating the inflammatory response and stenosis of coronary arteries [7]. Therefore, NLRP1 levels may become an important biomarker for predicting the severity and prognosis of PCHD. However, the predictive value of combined detection for PCHD and 2‐year long‐term prognosis is still unclear. In previous studies, PLR [8], MHR [9], and NLRP1 [10] have been confirmed to be associated with PCHD, and each can reflect the inflammatory state of the body and cardiovascular risk to a certain extent, but most of these studies explored the role of a single indicator in isolation. There is a lack of systematic studies on the combined detection of these three indicators in predicting the severity of PCHD and 2‐year long‐term prognosis of PCHD. In addition, there are no clear and in‐depth reports on the possible synergistic mechanism between them, as well as the clinical threshold and accurate predictive value when combined. The research hypothesis is as follows: In patients with PCHD, the combined detection of PLR, MHR, and NLRP1 can more accurately predict the severity of PCHD and 2‐year long‐term prognosis than the single index detection. Also, there is a potential synergistic mechanism between the three indicators, which can jointly provide more valuable risk assessment information for the clinic. It can provide more valuable risk assessment information for clinical practice, so as to help early identification of high‐risk patients, achieve targeted intervention, reduce the incidence and mortality of PCHD, and improve the quality of life of patients. This study aims to fill the gap in the existing literature and provide new ideas and methods for the clinical diagnosis and prognosis evaluation of PCHD.

## 2 Materials and Methods

### 2.1 General Materials

A total of 132 patients with PCHD who were examined in our hospital were retrospectively selected as the research objects for analysis, and the selected period was from February 2020 to January 2022. This sample size was chosen primarily on the basis of the following considerations: First, it would ensure sufficient data points for statistical analyses, particularly with respect to group comparisons (e.g., the groups with mild, moderate, or severe disease and the groups with favorable or unfavorable prognosis). Second, although the sample size was relatively limited, 132 patients were a relatively large sample in the retrospective study, limited by the amount of available data. It was sufficient to preliminarily explore the predictive value of MHR, PLR, and NLRP1 in the severity of PCHD and long‐term prognosis of PCHD.

**Figure 1:**
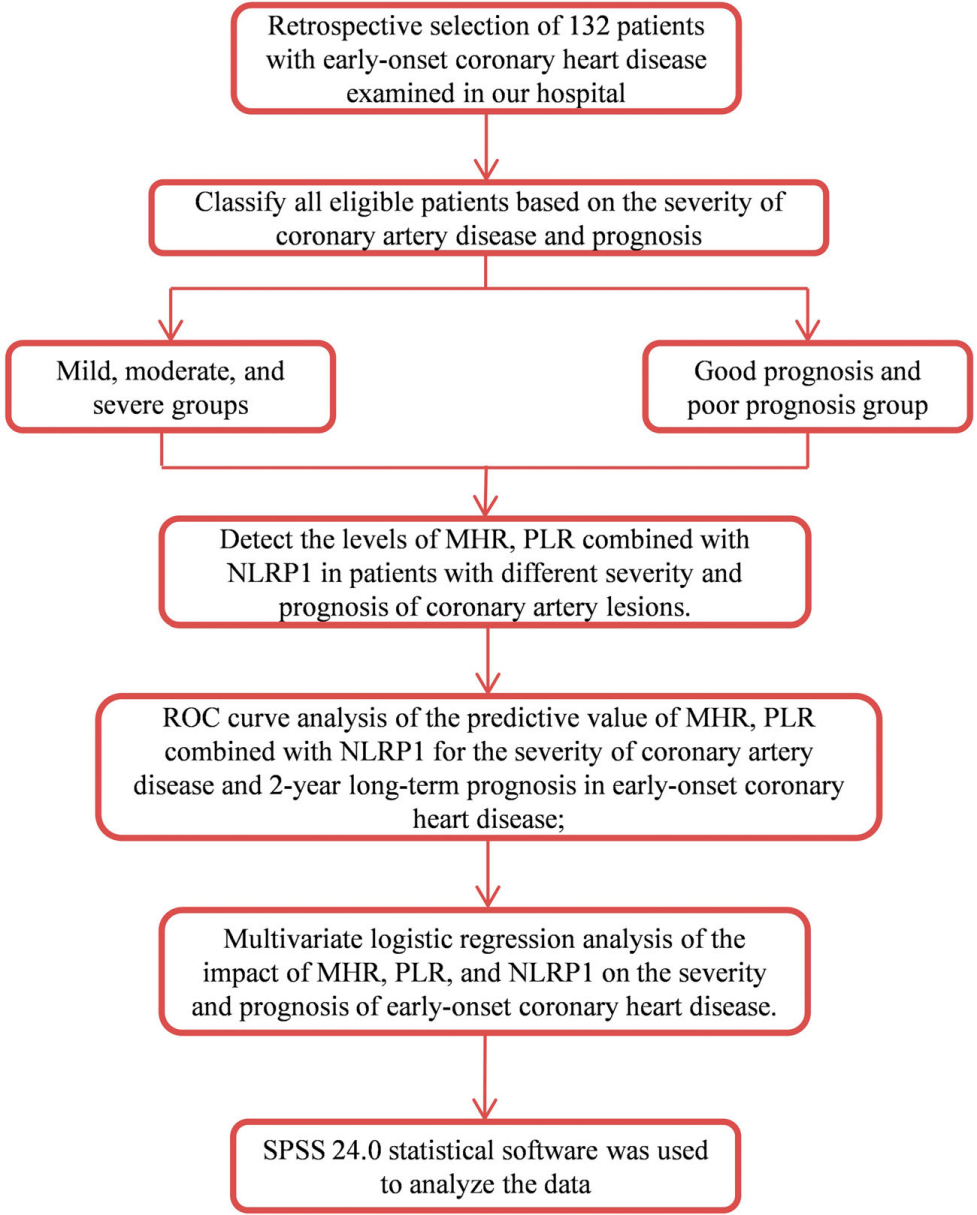
Study flowchart.

Inclusion criteria: (1) Patients met the diagnostic and treatment guidelines for PCHD [11]; (2) Coronary atherosclerosis was confirmed by coronary angiography; (3) Patients with complete clinical data; (4) Patients with clear cognitive functions. The specific definition criteria referred to the Mini‐Mental State Examination (MMSE) score [12], with MMSE score ≥ 24 points. Exclusion criteria: (1) Patients with liver and kidney dysfunction; (2) Patients with autoimmune diseases such as rheumatoid arthritis and systemic lupus erythematosus. The definition of immune diseases was based on the internationally recognized classification criteria of autoimmune diseases and meets the relevant diagnostic criteria formulated by the American College of Rheumatology (ACR) [13]; (3) Patients without coronary heart disease; (4) Patients undergone previous percutaneous coronary intervention (PCI). According to the severity of PCHD (Gensini score), all eligible patients were divided into mild group (< 30 points, *n* = 56), moderate group (30–60 points, *n* = 63), and severe group (> 60 points, *n* = 13). Among them, there were 26 males and 30 females in the mild group, aged 27–68 years, with an average age of 62.82 ± 9.63 years, 27 cases with a history of smoking and 31 cases with a history of hypertension. In the moderate group, there were 24 males and 39 females, aged 25–73 years, with an average age of 61.79 ± 8.55 years, 30 cases with smoking history, and 37 cases with hypertension history. In the severe group, there were 6 males and 7 females, aged 26–70 years, with an average age of 62.37 ± 11.18 years, 8 cases with a history of smoking, and 9 cases with a history of hypertension. Patients were followed for 2 years, and adverse cardiovascular events (including death, MI), myocardial revascularization (e.g., PCI or coronary artery bypass grafting), and unstable angina were measured according to previous studies [14]. The patients were divided into a good prognosis group (without adverse cardiovascular events, *n* = 96) and a poor prognosis group (with adverse cardiovascular events, *n* = 36). Among them, there were 36 males and 60 females in the good prognosis group, aged from 27 to 68 years with an average age of 63.84 ± 10.14 years. There were 10 males and 26 females in the poor prognosis group, aged from 27 to 68 years with an average age of 62.82 ± 9.63 years. There was no significant difference in general data among the groups (*p* > 0.05). The study flow was shown in Figure 1.

### 2.2 Indicators

#### 2.2.1 Peripheral Blood Sample Collection

Fasting venous blood of 4 mL was drawn from all subjects and placed in anticoagulant tubes. Serum and blood cells were separated by a low‐temperature high‐speed centrifuge (Shanghai Mituo Biotechnology Co. Ltd., Model: CF1524R) at a centrifugal force of 4500 r/min for 20 min. The supernatant was taken and placed in an ultra‐low temperature refrigerator at −80°C (Aiwantuo Life Science (Shanghai) Co. Ltd., Model: ULT‐U250) for subsequent detection. Quality control: During the sample collection process, aseptic operation standards were strictly implemented to avoid sample contamination. The operating state of the centrifuge was checked before each centrifugation to ensure that the centrifuge parameters were accurate. The ultra‐low temperature refrigerator regularly monitored and recorded the temperature to ensure that the sample was kept at a constant temperature and to prevent the sample from affecting the test results due to temperature fluctuations.

#### 2.2.2 MHR and PLR Detection

The monocyte count, lymphocyte count, platelet count, and high‐density lipoprotein level were detected by an automatic hematology analyzer (Jiashan Gasdyck Medical Equipment Co. Ltd., Model DH‐580), and the levels of PLR and MHR were calculated. Quality control: Calibrators were used to calibrate the automatic hematology analyzer before testing to ensure the accuracy of the instrument test results. Internal quality control was carried out after the device was turned on every day. High, medium, and low concentration quality control materials were used for testing to ensure that the test results were within the quality control range. Regularly participate in EQA activities and compare with other laboratories to verify the reliability of test results.

#### 2.2.3 NLRP1 Content Detection

The serum levels of NLRP1 were measured by enzyme‐linked immunosorbent assay in strict accordance with the instructions. Fasting venous blood of 4 mL was collected from all subjects and placed in an anticoagulant tube. Serum and blood cells were separated by a low‐temperature high‐speed centrifuge at 4500 r/min for 20 min. The supernatant was stored in an ultra‐low temperature refrigerator at −80°C until NLRP1 levels were measured. All reagents were diluted and configured according to the manufacturer's instructions. Standards or samples to be tested were added to 96‐well plates at 100 μL/well. After incubation, plate washing and other procedures, 90 μL of TMB substrate was added and incubated for 15 min in the dark. The reaction was subsequently terminated by adding termination solution. The absorbance values (OD values) of each well were read using a microplate reader at a wavelength of 450 nm, and the concentration of NLRP1 in the samples was calculated according to the standard curve. The concentration of NLRP1 was calculated by substituting the OD value of the sample into the formula based on the standard curve. To ensure the stability and reliability of the experimental results, the NLRP1 levels of each sample were detected by at least three repeated experiments, and the average value was taken as the final result. NLRP1 ELISA Kit was purchased from Shanghai Keaibo Biotechnology Co. Ltd. (Item No.: CB15809 Hu) and stored at 4°C. Quality control: During the experiment, the same batch of 96‐well plates was used to avoid the effect of batch differences on the experimental results. Blank, negative, and positive controls were set for each experiment to monitor the validity of the experiment. The plate washing process was carried out in strict accordance with the instructions to ensure that the plate was washed thoroughly and to avoid residual substances interfering with the test results. The microplate reader was preheated and calibrated before use to ensure the accuracy of wavelength and the reliability of absorbance detection.

### 2.3 Statistical Analysis

SPSS 24.0 statistical software was used for the data analysis. The measurement data, including the age, body mass index, and the levels of PLR, MHR, and NLRP1, etc., were represented by ($\bar{x}$ ± *s*). The measurement data between groups were compared using an independent sample *t*‐test. The measurement data among three groups were compared by one‐way analysis of variance, and pairwise comparisons between groups were conducted using the LSD‐*t*‐test. The enumeration data, including gender, smoking history, history of hypertension, etc., were represented as [cases (%)]. Intergroup comparison was conducted using*χ* ^2^ test. ROC curves were established to analyze the predictive value of MHR, PLR combined with NLRP1 for the severity of PCHD and 2‐year long‐term prognosis. Multivariate logistic regression analysis was used to analyze the impact of MHR, PLR, and NLRP1 on the severity and prognosis of PCHD. The detailed operation was as follows: First, all potential confounding variables were included as covariates in the initial model, and the backward method was used for variable screening. At each iteration, variables that did not contribute significantly to the model (*p* > 0.05) were removed according to the *p* value of the likelihood ratio test, until only variables that had a significant effect on the dependent variable were retained in the model. The final model was a multivariate Logistic regression model after adjusting for confounding factors. This model could evaluate the independent effects of MHR, PLR, and NLRP1 on the severity and prognosis of PCHD after adjusting for other factors. Through this method, the interference of confounding factors on the research results was effectively controlled, and the relationship between the research variables was more accurately revealed. *p* < 0.05 was considered statistically significant.

## 3 Results

### 3.1 Analysis of MHR, PLR, and NLRP1 in Patients With Different Degrees of PCHD

A total of 132 patients with PCHD were divided into mild group (56 cases), moderate group (63 cases), and severe group (13 cases) according to the severity of PCHD. Compared with the mild group, the levels of MHR, PLR, and NLRP1 in the moderate group and the severe group were significantly increased (*p* < 0.001, Table 1). In the mild group, MHR was 0.35 ± 0.04, PLR was 92.87 ± 23.14, and NLRP1 was 31.85 ± 9.24. The corresponding indexes were 0.51 ± 0.16, 114.76 ± 12.63, and 43.62 ± 10.69 in moderate group; 0.74 ± 0.12, 128.26 ± 25.31, and 57.55 ± 9.36 in the severe group. Compared with the moderate group, the increase of these three indicators in the severe group was more obvious, and the difference was highly statistically significant (*p* < 0.001, Table 1). This suggested that MHR, PLR, and NLRP1 levels increased with increasing severity of coronary artery lesions, indicating that they may be closely related to the progression of coronary artery lesions.

**Table 1:** Analysis of MHR, PLR, and NLRP1 in patients with different degrees of PCHD ($\bar{x}$ ± *s*).

Groups	Cases	MHR	PLR	NLRP1 (pg/mL)
The mild group	56	0.35 ± 0.04	92.87 ± 23.14	31.85 ± 9.24
The moderate group	63	0.51 ± 0.16***	114.76 ± 12.63***	43.62 ± 10.69***
The severe group	13	0.74 ± 0.12***, ^###^	128.26 ± 25.31***, ^###^	57.55 ± 9.36***, ^###^
*F*		64.61	28.59	42.97
*P*		< 0.001	< 0.001	< 0.001

*Note:* ***p < 0.001 compared with the mild group; ^###^ *p* < 0.001 compared with the moderate group.

### 3.2 The Effect of MHR, PLR, and NLRP1 on the Severity of PCHD by the Multivariate Logistic Regression Analysis

First, all potential confounding variables (such as treatment, comorbidities, weight, gender, smoking, etc.) were included in the model for preliminary analysis. According to the significance of the variables and the goodness‐of‐fit index of the model, insignificant variables were gradually eliminated until the best model was obtained. After adjusting for confounding factors such as treatment, comorbidities, weight, gender, and smoking, multivariate logistic regression analysis was performed with the severity of PCHD as the dependent variable (0 = mild‐to‐moderate, 1 = severe) and MHR, PLR, and NLRP1 as independent variables (all continuous variables). The results showed that MHR, PLR, and NLRP1 were all independent risk factors for the progression of PCHD to severe disease (*p* < 0.05, Table 2). These results indicated that MHR (OR = 1.243, 95% 1.058–1.462), PLR (OR = 4.430, 95% 1.655–11.856), and NLRP1 (OR = 1.052, 95% 1.011–1.094) were independent risk factors for the progression of PCHD to severe disease. That is, their elevation would significantly increase the risk of severe disease progression in patients.

**Table 2:** The impact of MHR, PLR, and NLRP1 on the severity of PCHD by multivariate logistic regression analysis.

Variables	*Β*	Wald *χ* ^2^	SE	*p*	OR value	95% CI
MHR	0.218	0.083	6.965	0.008	1.243	1.058 ~ 1.462
PLR	1.488	0.502	8.779	0.003	4.430	1.655 ~ 11.856
NLRP1	0.050	0.020	6.281	0.012	1.052	1.011 ~ 1.094

### 3.3 The Predictive Value of Combined Detection of MHR, PLR, and NLRP1 for the Severity of PCHD

**Figure 2:**
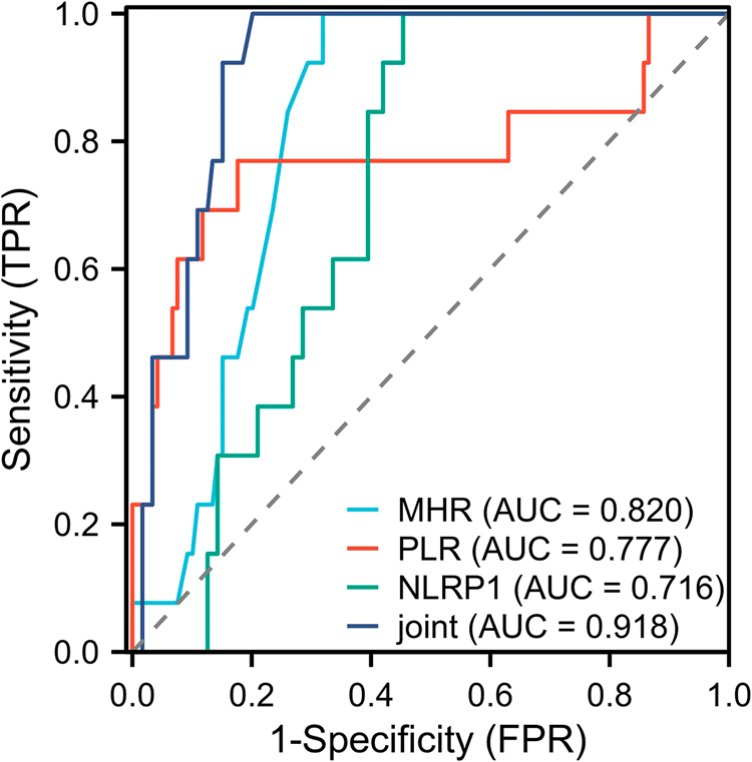
The predictive value of combined detection of MHR, PLR, and NLRP1 for the severity of PCHD. The ROC curve showed the predictive ability of MHR, PLR, and NLRP1 alone and in combination for the severity of PCHD. The larger the AUC was, the higher the predictive value was. The AUC of the combined detection was 0.918, indicating that it had high prediction accuracy. FPR: specificity, TPR: sensitivity.

To evaluate the predictive value of combined detection of MHR, PLR, and NLRP1 for coronary severity, ROC curves were established. As shown in Figure 2, the AUC of MHR, PLR, and NLRP1 alone and in combination were 0.820, 0.777, 0.716, and 0.918, respectively. The AUC of combined detection was significantly higher than that of individual detection, indicating that combined markers showed superior predictive accuracy (*p* < 0.05). Table 3 details the sensitivity, specificity, cutoff values, Youden's index, and 95% confidence intervals of each index individually and in combination. This result indicated that combined detection of MHR, PLR, and NLRP1 could more effectively predict the severity of PCHD, and provide a more reliable basis for clinical decision‐making.

**Table 3:** The predictive value of combined detection of MHR, PLR, and NLRP1 for the severity of PCHD.

Indicators	AUC	Sensitivity (%)	Specificity (%)	Cutoff value	Youden's index	*p*	95% CI
MHR	0.820	90.31	72.49	0.59	0.628	< 0.05	0.745–0.894
PLR	0.777	76.92	82.35	118.60	0.593	< 0.05	0.596–0.958
NLRP1	0.716	83.25	62.33	49.16 pg/mL	0.456	< 0.05	0.621–0.810
Combined detection	0.918	91.42	87.26	—	0.787	< 0.05	0.868–0.968

### 3.4 Comparison of MHR, PLR, and NLRP1 Levels in Patients With Different Prognoses

By comparing the MHR, PLR, and NLRP1 levels of patients in the good prognosis group with those in the poor prognosis group, we found significant differences. Specifically, the levels of MHR, PLR, and NLRP1 in the poor prognosis group showed a significantly higher trend than those in the good prognosis group, and these differences were statistically significant (*p* < 0.001, Table 4).

**Table 4:** Comparison of MHR, PLR, and NLRP1 content in patients with different prognoses ($\bar{x}$ ± *s*).

Groups	Cases	MHR	PLR	NLRP1 (pg/mL)
Good prognosis group	96	0.68 ± 0.25	121.82 ± 45.58	48.36 ± 6.38
Poor prognosis group	36	0.98 ± 0.34	147.42 ± 42.42	63.42 ± 7.46
*t*		5.539	2.927	11.522
*p*		< 0.001	0.004	< 0.001

### 3.5 The Effect of MHR, PLR, and NLRP1 on the Prognosis of PCHD by the Multivariate Logistic Regression Analysis

After adjusting for confounding factors such as treatment, comorbidities, body weight, gender, and smoking, multivariate logistic regression analysis was performed with the prognosis of PCHD as the dependent variable (0 = good prognosis, 1 = poor prognosis) and MHR, PLR, and NLRP1 as independent variables (all continuous variables). The results showed that MHR, PLR, and NLRP1 were independent risk factors for poor prognosis in patients with PCHD (*p* < 0.05, Table 5). These results suggested that MHR, PLR, and NLRP1 were important evaluation indicators for predicting the prognosis of PCHD, even after considering other possible influencing factors.

**Table 5:** The impact of MHR, PLR, and NLRP1 on the prognosis of PCHD by multivariate logistic regression analysis.

Variables	*β*	Wald *χ* ^2^	SE	*p*	OR	95% CI
MHR	0.802	6.391	0.317	0.011	2.230	1.025 ~ 5.261
PLR	0.823	6.683	0.318	0.010	2.277	1.115 ~ 3.897
NLRP1	0.728	4.961	0.327	0.026	2.071	1.025 ~ 4.897

### 3.6 The Predictive Value of Combined Detection of MHR, PLR, and NLRP1 for the Prognosis of Patients

**Figure 3:**
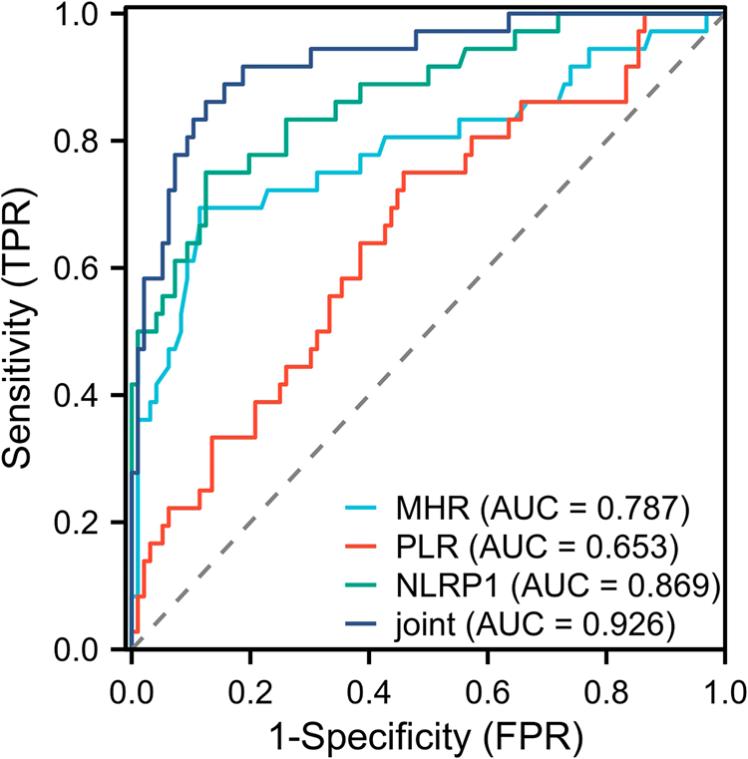
Predictive value of MHR, PLR, and NLRP1 combined detection for 2‐year long‐term prognosis of patients. ROC curve showed the predictive ability of MHR, PLR, and NLRP1 alone and in combination for 2‐year long‐term prognosis in patients with PCHD. The AUC of combined detection was 0.926, indicating that it had high accuracy in predicting the long‐term prognosis of patients. FPR: specificity, TPR: sensitivity.

To evaluate the predictive value of combined detection of MHR, PLR, and NLRP1 for 2‐year long‐term prognosis of patients, ROC curves were established. As shown in Figure 3, the AUC of MHR, PLR, and NLRP1 alone and in combination were 0.787, 0.653, 0.869, and 0.926, respectively. The AUC of combined detection was significantly higher than that of individual detection, indicating that combined markers showed superior predictive accuracy (*p* < 0.05). Table 6 details the sensitivity, specificity, cutoff values, Youden's index, and 95% confidence intervals of each index individually and in combination. These results indicated that combined detection of MHR, PLR, and NLRP1 could more effectively predict the 2‐year long‐term prognosis of patients with PCHD, and provide a more reliable basis for clinical decision‐making.

**Table 6:** Predictive value of MHR, PLR, and NLRP1 combined detection for 2‐year long‐term prognosis of patients.

Indicators	AUC	Sensitivity (%)	Specificity (%)	Cutoff value	Youden's index	*p*	95% CI
MHR	0.787	69.44	88.54	0.94	0.580	< 0.05	0.685–0.888
PLR	0.653	75.00	54.17	122.73	0.291	< 0.05	0.548–0.758
NLRP1	0.869	75.00	87.50	55.59 pg/mL	0.625	< 0.05	0.797–0.940
Combined detection	0.926	86.11	87.50	—	0.736	< 0.05	0.875–0.976

## 4 Discussion

In recent years, an increasing number of studies have highlighted the importance of endothelial dysfunction in cardiovascular diseases. Prabhahar et al. [15] pointed out that endothelial dysfunction is not only closely related to cardiovascular complications after transplantation, but also significantly related to the long‐term survival rate of patients in the kidney transplant population. This indicates that endothelial dysfunction is not only a marker of cardiovascular disease, but also an important factor affecting its prognosis. Similarly, the review article by Medina‐Leyte et al. [16] comprehensively elaborated the relationship between endothelial dysfunction, inflammation and coronary artery disease. The study pointed out that endothelial dysfunction accelerated the process of atherosclerosis by promoting inflammatory response, increasing vascular permeability, affecting platelet function, and other mechanisms. It also increases the risk of adverse cardiovascular events.

The pathogenesis of PCHD is complex. Smoking, body mass index, dyslipidemia, hypertension, diabetes, and hyperhomocysteinemia are important risk factors affecting the occurrence and development of coronary heart disease. A variety of factors work together to cause the development of coronary heart disease [17]. Among them, inflammation and lipids play an important role in the formation of atherosclerosis and thrombosis [18]. MHR is the ratio of monocytes to high‐density lipoprotein. Monocytes are the main source of anti‐inflammatory substances in the process of atherosclerosis. They can release inflammatory factors and pro‐oxidation factors into the scaffold and interact with platelets and smooth muscle cells, thereby causing the activation of inflammatory response and aggravating the development of the disease [19]. However, high‐density lipoprotein can promote the efflux of cholesterol in cells, and has a certain protective effect on the oxidation of low‐density lipoprotein. It can also directly act on monocytes and inhibit inflammatory response [20]. It has been found that MHR is significantly increased in patients with PCHD, and MHR levels increase significantly with the increase of the severity of PCHD [21, 22]. Basic research has confirmed that the activation of monocytes is an important step in initiating the process of atherosclerosis [23]. Monocytes interact with the activated vascular endothelium to promote the expression of adhesion molecules and pro‐inflammatory factors. It then triggers an inflammatory cascade reaction and induces monocytes to differentiate into macrophages. Subsequently, macrophages phagophagy oxidized low density lipoprotein and differentiate to form lipid foam, initiating atherosclerosis. Therefore, MHR is closely related to the inflammatory state of the body, reflecting the balance between atherogenic and anti‐atherogenic. The above studies suggest that MHR can be used as a powerful marker for assessing the severity of PCHD.

PLR is the ratio of platelets to lymphocytes. When arteriosclerosis occurs, monocytes and lymphocytes migrate to the arterial endothelium. The number of lymphocytes is significantly reduced when plaque is formed in the blood. It has been reported that low lymphocyte count significantly increases the risk of cardiovascular disease [24]. In the process of inflammation and thrombosis, platelet is an important component. The increase of platelet count may promote platelet activation and increase the release of inflammatory mediators, leading to the inflammatory process that is harmful to the body [25]. A number of studies have found that PLR levels increase significantly with the increase of the severity of PCHD, and its level is closely related to the prognosis of patients [26, 27]. NLPR1 is an inflammasome. The study has found that the level of NLRP1 is related to the severity of coronary artery stenosis in patients with PCHD. It can activate Caspase‐1 and promote the secretion of inflammatory cytokines, aggravate the inflammatory response, and lead to coronary artery stenosis [28]. NLRP1 inflammasome can aggravate the damage of myocardial cells and promote the expansion of MI by inducing the release of inflammatory factors such as IL‐1β. At the same time, it can also induce the damage of vascular endothelial cells and promote the formation of vascular stenosis and atherosclerosis [29]. In this study, MHR, PLR, and NLRP1 levels in patients with different severities of PCHD and different prognoses were analyzed. The results showed that MHR, PLR, and NLRP1 levels in patients with severe disease and poor prognosis were significantly higher than those in patients with mild disease and good prognosis. Therefore, the changes of MHR, PLR, and NLRP1 can be used to evaluate the severity and prognosis of patients with PCHD. In evaluating the severity of PCHD, MHR, PLR, and NLRP1 can be combined with traditional risk factors (such as blood lipids, blood pressure, etc.) to construct a more comprehensive risk assessment model. When the level of these biomarkers is increased, it can prompt clinicians to pay attention to the possibility of coronary artery disease even if the patient has not yet developed obvious clinical symptoms. Therefore, preventive measures, such as lifestyle modification and intensive drug treatment, should be taken more actively to delay the progression of the disease. In clinical practice, for those patients at borderline risk or with atypical symptoms, the results of these biomarkers can assist doctors to make more accurate diagnostic decisions and avoid missed diagnosis or misdiagnosis.

Compared with the traditional inflammatory markers that have been confirmed, NLR has attracted much attention in cardiovascular disease research as a commonly used inflammatory indicator. The study by Tudurachi et al. [30] pointed out that NLR levels can change significantly in patients with acute MI, with some specificity in young patients. In an observational study of patients with ST‐segment elevation myocardial infarction (STEMI), Sharma et al. [31] point out that NLR is an effective preliminary prognostic marker and can predict the clinical outcome of patients. There are similarities to the association of MHR with PCHD in the present study. However, NLR mainly reflects the balance changes of neutrophils and lymphocytes, while MHR focuses on the relationship between monocytes and high‐density lipoprotein. They can reflect the inflammatory state from different perspectives. When assessing the severity of PCHD, NLR may focus more on the degree of systemic inflammatory response, while MHR focuses more on the local inflammatory microenvironment changes related to atherosclerosis. FAR is also an inflammatory marker that has been studied more in recent years. Xie et al. [32] found in their study that FAR was associated with long‐term mortality in patients with PCHD under different glucose metabolism states. Makkar et al. [33] showed that FAR had certain value in judging angiographic severity and prognostic outcome in patients with acute coronary syndrome. Compared with FAR, which mainly reflects the inflammation related to coagulation and nutritional status, PLR in this study mainly reflects the relationship between platelets and lymphocytes in the process of inflammation and thrombosis. The mechanism and focus of inflammation are different between the two. In predicting the prognosis of patients with PCHD, FAR may be more affected by the overall nutrition and coagulation function of patients, while PLR more directly reflects the relationship between immune inflammatory response and platelet function. The study by Khandelwal et al. [34] explored the relationship between CRP and the clinical spectrum of PCHD and the perioperative outcomes of PCI. In the process of the occurrence and development of PCHD, CRP reflects the changes in the overall inflammatory level, while NLRP1 reveals the initiation and amplification mechanism of inflammatory response from the cellular level. They complement each other and provide information for the assessment of PCHD. Compared with previous studies, the combined detection of MHR, PLR, and NLRP1 in this study has high value in predicting the severity of PCHD and 2‐year long‐term prognosis, which is consistent with some previous studies to a certain extent.

In addition, this study found that the AUC for predicting the severity of PCHD using MHR, PLR, and NLRP1 alone and in combination was 0.820, 0.777, 0.716, and 0.918, respectively. This indicated that these three indicators had certain value in evaluating the severity of PCHD. In Kundi's study [35], patients with high SYNTAX scores had significantly higher MHR and could be used to predict the prognosis of patients with PCHD with a sensitivity of 66% and a specificity of 65.1%. One study [36] also found that PLR was negatively correlated with ejection fraction in patients with heart failure. The AUC of PLR in predicting the occurrence of heart failure was 0.760, which confirmed the practicability of PLR in evaluating the clinical status of elderly patients with congestive heart failure. It has been found that serum NLRP1 levels increase with the number of diseased coronary branches according to the number of diseased coronary branches [37]. Serum NLRP1 levels were significantly higher in patients with multivessel disease than in controls and patients with single‐vessel disease, and serum NLRP1 levels were positively correlated with the severity of coronary atherosclerosis, consistent with the results of this study. The AUC for predicting prognosis using MHR, PLR, and NLRP1 alone and in combination were 0.787, 0.653, 0.869, and 0.926, respectively. The combination of the three indicators had higher clinical value in the evaluation of 2‐year long‐term prognosis of patients. It can lay a clinical foundation for the later study of the role and molecular mechanism of MHR, PLR, and NLRP1 in cardiac remodeling and heart failure in patients with PCHD, and has strong feasibility in theory. Clinical threshold is of key significance in practical application. When MHR is > 0.59, its accuracy in predicting coronary artery severity is relatively high, and clinicians can make a preliminary risk assessment of patients based on this. For PLR, the cutoff value of 118.60 can be used as an important reference index to judge the severity of PCHD. If the value is higher than this, the patient may have a serious risk of PCHD. For NLRP1, the threshold of 49.16 pg/mL similarly provides valuable information for clinical judgment. The cutoff value of MHR is 0.94. When the MHR is higher than this value, the risk of poor 2‐year prognosis increases. So, it is necessary to pay close attention to the patient's condition and strengthen the follow‐up. The cutoff values of PLR (122.73 pg/mL) and NLRP1 (55.59 pg/mL) also provide quantitative reference for clinicians to judge the prognosis of patients. By monitoring and analyzing these thresholds, combined with the clinical manifestations and other examination results of patients, the prognosis of patients can be more comprehensively evaluated, the treatment strategy can be adjusted in time, and the survival rate and quality of life of patients can be improved. Traditional coronary angiography is the “gold standard” for the diagnosis of PCHD. Although it can visually display the anatomical structure and lesions of coronary arteries, it is an invasive examination with certain operational risks, such as bleeding, infection, and vascular injury. Moreover, it is not suitable for all patients, especially for some patients with poor physical conditions or contraindications to angiography [38]. The electrocardiogram examination is simple and cheap, and it has certain value in detecting myocardial ischemia. However, the sensitivity of detecting early coronary artery lesions is low, and the evaluation of the severity of coronary artery lesions is relatively limited. The electrocardiogram of some patients with PCHD may only show nonspecific changes, which may easily lead to missed diagnosis [39]. Compared with these traditional diagnostic tools, the combined detection of MHR, PLR, and NLRP1 as a noninvasive detection method has the advantages of convenience and strong repeatability. It can reflect the coronary artery lesions from multiple dimensions, such as inflammatory response and cellular level, and provide more comprehensive information for clinical practice. Although the current AUC of combined detection shows high predictive accuracy, it is not a complete replacement for traditional diagnostic methods. In practical clinical application, if the combined detection is combined with traditional methods, such as clinical symptoms, electrocardiogram, and coronary angiography, the advantages are complementary. It is expected to further improve the accuracy of PCHD severity assessment in patients with PCHD, and to provide a more comprehensive and reliable basis for clinical decision‐making. Regarding the statistical power of the sample size, a preliminary assessment was performed. Considering that the main purpose of this study was to explore the predictive value of MHR and PLR combined with NLRP1, rather than to establish a large‐scale epidemiological model, the sample size of 132 patients was acceptable for the preliminary exploratory study. The high AUC values obtained by ROC curve analysis (0.918 for the prediction of coronary severity and 0.926 for the prediction of long‐term prognosis) indicate the good predictive performance of these biomarkers with the available sample size. However, to more accurately assess the clinical utility of these markers, future studies should consider expanding the sample size and validating them in a broader patient population.

The combined detection of biomarkers in this study has unique advantages as a noninvasive method. Compared with the commonly used electrocardiogram, the electrocardiogram has a certain value in detecting myocardial ischemia, but it is relatively limited in the evaluation of early coronary artery lesions and prognosis. The combined detection of MHR, PLR, and NLRP1 can reflect coronary artery lesions from multiple dimensions, such as inflammatory response and cellular level, and its higher AUC value shows that it may have more potential in prediction accuracy. For another example, compared with the assessment of clinical symptoms and medical history alone, the biomarkers in this study can provide more objective quantitative indicators. Clinical symptoms and medical history assessment are easily affected by the subjective feelings of patients and the differences in doctor's experience, and the test results of these biomarkers are relatively stable and highly reproducible. In practical clinical application, if the combined detection is combined with traditional methods such as clinical symptoms and electrocardiogram, it is expected to further improve the accuracy of assessing the severity of PCHD and long‐term prognosis in patients with PCHD. It can provide a more comprehensive and reliable basis for clinical decision‐making, so as to better guide the formulation of personalized treatment plans and improve the clinical outcomes of patients.

## 5 Conclusion

In general, this study investigated the predictive value of PLR, MHR, and NLRP1 in the severity of PCHD and 2‐year long‐term prognosis of PCHD. The data of 132 patients were retrospectively analyzed. It was found that the levels of MHR, PLR, and NLRP1 in the moderate and severe groups were significantly higher than those in the mild group, and the severe group was higher than the mild group. ROC curve showed that the combined detection had the highest predictive value (AUC = 0.918). The above indicators in the poor prognosis group were significantly higher than those in the good prognosis group, and the combined detection had the highest predictive value for 2‐year long‐term prognosis (AUC = 0.926). Logistic regression analysis showed that all three were independent risk factors for disease progression and poor prognosis. It has found that these new inflammatory markers provide new diagnostic tools for clinical practice, and combined detection can improve the prediction accuracy and help to develop more reasonable treatment plans, especially for high‐risk patients.

### 5.1 Limitations

This study used a single‐center design, and the samples were all from the same hospital, which may lead to sample selection bias and affect the generalization of the results. Future studies should consider multicenter and large sample validation. At the same time, this study is a retrospective study based on existing medical records, which may have incomplete information or inaccurate records, and cannot control all confounding factors or establish causality. Prospective studies and randomized controlled trials (RCTS) can more accurately evaluate the relationship between MHR, PLR, NLRP1, and PCHD. The single‐center design and retrospective analysis may affect the representativeness of the sample and the reliability of the results, which should be interpreted in combination with other studies. In order to overcome the possible sample selection bias caused by the single‐center design, future studies should consider recruiting patients from multiple medical institutions at different levels in different regions and conduct multicenter studies. This will help to increase the diversity and representativeness of the sample and improve the generality and reliability of the findings. In order to more accurately evaluate the relationship between MHR, PLR, NLRP1, and PCHD, prospective RCTS should be considered in future studies. RCTS can control confounding factors, establish causality, and provide a higher level of evidence support. To address the possible problems of incomplete information or inaccurate records in retrospective studies, future studies should strengthen data quality control and validation measures.

#### 5.1.1 Novelty and Clinical Relevance

##### 5.1.1.1 Novelty

In this study, PLR, MHR, and NLRP1 were combined to predict the severity of PCHD and 2‐year long‐term prognosis of PCHD, which filled the research gap in this field. ROC curve analysis and multivariate logistic regression analysis confirmed the high value of combined detection of these three indicators in predicting the condition and prognosis of PCHD, which provided a new noninvasive diagnostic idea for clinical practice.

###### 5.1.1.1.1 Clinical Relevance

This study has important clinical implications. On the one hand, combined detection of MHR, PLR, and NLRP1 can help to identify patients with high‐risk PCHD early and provide a basis for the formulation of personalized treatment plans. On the other hand, early intervention, such as intensive drug therapy or early coronary intervention surgery, can significantly reduce the risk of adverse cardiovascular events, improve the prognosis of patients, and improve the quality of life of patients. On the other hand, early intervention, such as intensive drug therapy or early coronary intervention surgery, can significantly reduce the risk of adverse cardiovascular events, improve the prognosis of patients, and improve the quality of life of patients. In addition, this study provides a new perspective and clues for further exploring the pathogenesis and biomarkers of PCHD.

## Author Contributions

**Mengyun Zhu:** investigation, methodology, writing – original draft. **Jianying Shen:** data curation, formal analysis, methodology. **Weijing Liu:** investigation, software, supervision, validation, visualization. **Hui Sun:** project administration, resources, software. **Yawei Xu:** conceptualization, investigation, resources, writing – review and editing. All authors read and approved the final manuscript.

## Ethics Statement

All procedures performed in studies involving human participants were in accordance with the ethical standards of the institutional and/or national research committee and with the 1964 Helsinki Declaration and its later amendments or comparable ethical standards. All procedures performed in studies were in accordance with the ethical standards of the Ethics Committee of Shanghai Tenth People's Hospital (2020‐KN16‐03).

## Consent

Written Informed consent was obtained from all individual participants included in the study. The patients participating in the study all agree to publish the research results.

## Conflicts of Interest

The authors declare no conflicts of interest.

## Data Availability

The authors confirm that the data supporting the findings of this study are available within the article. The data sets used and/or analyzed during the current study are available from the corresponding author on reasonable request.
